# Impact of the Five-Year Intervention of an Antimicrobial Stewardship Program on the Optimal Selection of Surgical Prophylaxis in a Hospital without Antibiotic Prescription Restrictions in Costa Rica: A Retrospective Study

**DOI:** 10.3390/antibiotics12111572

**Published:** 2023-10-28

**Authors:** José Pablo Díaz-Madriz, Esteban Zavaleta-Monestel, Jorge Arturo Villalobos-Madriz, Carolina Rojas-Chinchilla, Priscilla Castrillo-Portillo, Alison Meléndez-Alfaro, Ana Fernanda Vásquez-Mendoza, Gabriel Muñoz-Gutiérrez, Sebastián Arguedas-Chacón

**Affiliations:** 1 Pharmacy Department, Hospital Clínica Bíblica, San José 10104, Costa Rica; jdiazm@clinicabiblica.com (J.P.D.-M.); jvillalobosma@clinicabiblica.com (J.A.V.-M.); crojasc@clinicabiblica.com (C.R.-C.); sarguedas@clinicabiblica.com (S.A.-C.); 2 Faculty of Pharmacy, Universidad de Ciencias Médicas, San José 10108, Costa Rica; castrillopri@gmail.com (P.C.-P.); alismelendez24@gmail.com (A.M.-A.); 3 Antimicrobial Stewardship Program, Hospital Clínica Bíblica, San José 10104, Costa Rica; avasquez@clinicabiblica.com (A.F.V.-M.); gmunozg@clinicabiblica.com (G.M.-G.)

**Keywords:** antibiotic prophylaxis, surgery department, hospital, antimicrobial stewardship

## Abstract

This study aims to characterize the impact of the implementation of an antimicrobial stewardship program (AMS) on the optimal selection of surgical antibiotic prophylaxis in adult patients. This is a retrospective quasi-experimental study that compared the selection and duration of antibiotics for all surgical prophylaxis prescriptions over six months, both before (pre-AMS) and after a five-year intervention of AMS (post-AMS). In addition, data related to the consumption of antibiotics, adverse drug reactions, and surgical site infections throughout the years of the intervention were analyzed. The rate of appropriate selection of antibiotic prophylaxis in surgical procedures improved to 80% during the post-AMS period. The percentage of optimal duration increased from 69.1% (*N* = 1598) in the pre-AMS period to 78.0% (*N* = 841) in the post-AMS period (*p* < 0.001). The consumption of ceftriaxone significantly decreased, while the use of cefazolin increased more than nine times. No severe adverse reactions or increases in surgical site infections were detected after the intervention. The implementation of an AMS in the surgical ward demonstrated a trend towards a positive overall impact on the selection and duration of prophylactic antibiotics for surgery, with positive results also observed in other variables associated with the prescription of these antibiotics.

## 1. Introduction

Antibiotics represent one of the most significant contributions to public health, as they prevent and treat life-threatening infectious diseases. However, their excessive and indiscriminate use has led to several problems, including an alarming increase in antibiotic resistance, a reduction in the availability of effective antibiotics against multi-resistant pathogens, and a rise in healthcare costs [1,2].

The Latin American Network for Antimicrobial Resistance Surveillance (ReLAVRA) has reported a sustained increase in antimicrobial resistance over the last decade. This increase exhibits wide heterogeneity in its magnitude; for example, resistance to meropenem in *K. pneumoniae* ranged from 10% to 69% across various countries in this region [3,4].

Surgical wards represent a critical area where the use of antimicrobials must be optimized. Around 14–17% of healthcare-associated infections worldwide consist of surgical site infections, and surgical antibiotic prophylaxis reduces the risk across different procedures [5]. Approximately 15% of antibiotic consumption in hospitals is used for this purpose [6]. Despite this, it has been found that up to 50% of antibiotic prescriptions in hospitals are suboptimal [7]. Hospitals implementing clinical guidelines for appropriate prophylaxis have identified that approximately 40% of patients undergoing elective surgery did not receive an antibiotic that adhered to these guidelines [8]. Several studies have examined the utilization of antibiotics in Latin America, and found that up to 13.3% of prescribed antibiotics are for surgical prophylaxis. Furthermore, surgical site infections are the second most common healthcare-associated infection (HAI) in the region [9,10].

Regarding the duration of prophylaxis, it is generally recommended that surgical antibiotic prophylaxis should be initiated within 1 h before incision and discontinued within 24 h postoperatively for most procedures [11]. Conversely, a correlation exists between prolonged use and a heightened risk of preventable adverse events, such as acute kidney injury (AKI) and *Clostridioides difficile* (CD) infection [12].

Antimicrobial stewardship programs (AMSs) represent a strategy with favorable results in optimizing the use of antibiotics. The implementation of AMSs in Latin America has been challenging due to several obstacles [10,13]. These include the lack of strong governmental leadership to support policies and financing, inappropriate indicators for measuring results, difficulties in incorporating technological systems such as digital records, deficiencies in hospital infrastructure, lack of education on habit changes, physician behavioral factors such as power differentials with other healthcare professionals (e.g., pharmacists) and uncertainty avoidance, non-adherence to treatment protocols, and a shortage of trained clinical pharmacists dedicated to AMS [14,15,16,17,18,19].

Due to an upward trend in the use of broad-spectrum antibiotics, the clinical administration of the Hospital Clínica Bíblica (HCB) approved the implementation of AMS in June 2015. This initiative was proposed by the Department of Pharmacy, led by the hospital clinical pharmacist, and supported by other healthcare professionals, such as an infectious disease physician, a clinical microbiologist who specializes in bacteriology, and the nurse who oversees the Infection Control Committee. The AMS model chosen for implementation by the hospital does not impose restrictions on antimicrobial prescription. A handshake stewardship methodology was used, in which a change in prescription habits is sought through daily prescription monitoring, education, and direct communication with physicians [20].

The AMS’s first project on this topic was in gynaecological surgery, which achieved positive results. A similar approach was continued for the rest of the group of surgical specialties in a progressive five-year program [21]. The strategy used was based on the model of continuous quality improvement and the cycle of continuous improvement [22]. The clinical guideline for antibiotic prophylaxis for surgery in adults was created based on the Bratzler et al. guideline [23]. This was accompanied by group and individualized education, dissemination of educational materials, monitoring of results using performance indicators, and prospective and retrospective audits with individualized feedback. It is important to note that Costa Rica does not have national guidelines for the use of antibiotics for treatment or prophylaxis.

The objective of this study is to evaluate the effects of implementing an AMS on the prescription of surgical antibiotic prophylaxis for surgeries conducted at HCB, located in San José, Costa Rica. Additionally, this research aims to compare other variables related to the five-year intervention period, including consumption patterns of frequently used preoperative antibiotics and patient safety outcomes.

## 2. Results

### 2.1. Comparison of the Percentages of Optimal Selection and Duration of the Surgical Prophylactic Scheme, before and after the Implementation of the AMS

Following a five-year intervention with an AMS, we observed a marked improvement in the appropriate selection of drugs for surgical prophylaxis, with the optimal rate rising significantly (*p* < 0.001) from 20% (*N* = 325) to 80% (*N* = 671). Additionally, the percentage of optimal treatment duration also demonstrated a significant increase (*p* < 0.001), rising from 69% (*N* = 1104) to 78% (*N* = 656).

Table 1 displays the surgical procedures conducted and the optimal selection of antibiotic prophylaxis during both the pre-AMS and post-AMS periods. It is important to note that the number of procedures performed in the hospital decreased in the post-AMS period. This situation might be attributable to patients now having access to an increased number of affordable options in the private sector. Additionally, if their economic situation prevents them from utilizing private medical services, they have the option to use the public health system.

Upon analyzing the data presented in Table 1, we found that during both periods, cesarean delivery was the most frequently conducted medical procedure, followed by orthopedic procedures. These two groups of surgeries collectively represent over 50% of the total surgical cases included. The data showed an enhancement in those procedures, with respective improvements of 93.3% and 52.8% in the selection of appropriate antibiotics for surgical prophylaxis. Similar positive trends occurred in most procedures examined. Conversely, certain groups of procedures did not exhibit a change, as observed in head and neck surgeries.

During the pre-AMS period, antibiotics were administered for more than 24 h in 494 (30.91%) out of 1598 procedures conducted. In the post-AMS period, out of the 841 procedures conducted, 191 (22.71%) were administered antibiotics for more than 24 h. This change represents a significant improvement in the duration of the prophylaxis. Cesarean deliveries, orthopedic procedures, and hysterectomies represent the prescriptions where improvements were significant (Table 2).

Ceftriaxone was the most frequently used antibiotic during the pre-AMS period, and its usage underwent a reduction of 60.5% in the post-AMS period. On the other hand, the most used antibiotic during the post-AMS period was Cefazolin. Table 3 displays the primary preoperative antibiotics administered during both the pre-AMS and post-AMS periods.

### 2.2. Changes in the Consumption of Antimicrobials from the Implementation of the AMS

**Figure 1:**
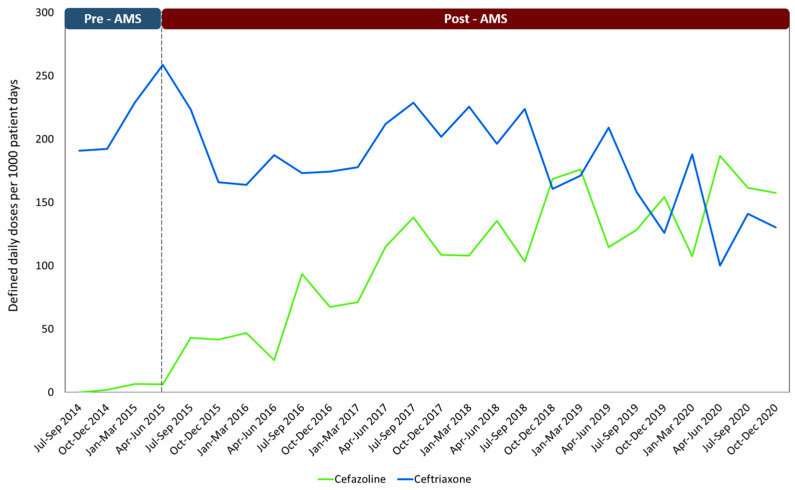
Consumption of the antibiotics most widely used in surgical prophylaxis using the DDD/1000 patient days method through the established periods. Note: AMS: Antimicrobial stewardship program.

Table 4 displays the comparison between the average hospital consumption of Ceftriaxone and Cefazolin for both periods, and Figure 1 illustrates the consumption patterns through time of these two medications from July 2014 to December 2020.

### 2.3. Adverse Effects Associated with Medications and Surgical Wound Infections

**Figure 2:**
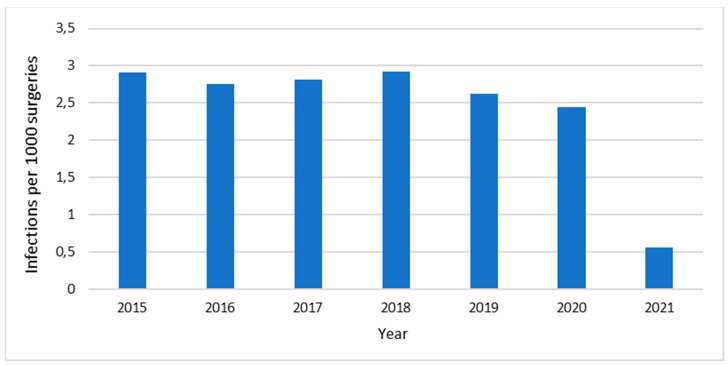
Surgical site infections (infections/1000 surgeries) at Hospital Clínica Bíblica, 2015–2021.

Figure 2 shows the incidence of surgical site infections (Infections/1000 surgeries) at HCB in the years 2015–2021. The number of infections has sustained stability, exhibiting no inclination towards escalation, and decreasing in 2021. The hospital’s pharmacovigilance databases were reviewed, and no adverse effects associated with the use of antimicrobials were reported during the study period.

## 3. Discussion

The current study demonstrates that the implementation of AMS may have resulted in a significant shift in prescription patterns, as evidenced by improvements in both the selection and duration of surgical antibiotic prophylaxis.

In Latin American hospitals, compliance with the clinical guidelines for antibiotic prophylaxis, as determined by point prevalence surveys, has shown room for improvement, with an adherence rate of approximately 44.3% (ranging from 28.5% to 54%). This is mainly characterized by issues in the selection and duration of antibiotics [10]. The data from this intervention show that after AMS implementation, the percentage of appropriate drug selection increased from 21% to 80%, while the appropriate duration increased from 69% to 78% in a statistically significant manner.

Cesarean deliveries and orthopedic surgeries are the procedures most frequently performed at our hospital. Notably, there has been a significant improvement in the selection and duration of antibiotic prophylaxis for these surgeries. Numerous studies from various regions across the globe have documented positive results in terms of prophylactic drug selection for gynecologic, obstetric, and orthopedic procedures after implementing an AMS in medical centers. These outcomes include cost reduction, enhancements in antibiotic prescription practices, and tangible clinical benefits for patients [24,25,26,27].

Our results indicate no improvement in the appropriate use of antibiotics for head and neck surgeries. In this type of surgery, it is necessary to carry out an additional intervention with the otolaryngologists, following the same intervention approach as with other specialties. These physicians are prescribing surgical prophylaxis in clean procedures that do not routinely require antibiotics, such as septoplasty, functional endoscopic sinus surgery (FESS), thyroidectomy, and tonsillectomy. Furthermore, they are extending the use of antibiotics, which has not been shown to provide benefits to patients [23]. Additionally, suboptimal combinations of antibiotics, such as Ceftriaxone and Clindamycin, have been observed in other cases. This issue requires the implementation of different actions from the AMS, in line with the hospital’s clinical guidelines [8,28].

The study found an optimal discontinuation rate of 78% for antibiotics. While a significant improvement was made, additional efforts are necessary to reduce the use of antibiotics even further in urologic, plastic surgery, and other procedures performed in this center. It is still a common practice among many surgeons to continue prophylactic intravenous or oral antibiotics for more than 24 h after the procedure. However, it is well documented that this practice does not affect subsequent surgical site infections and could increase the incidence of adverse effects [8,29]. In the region, it has been ascertained that the primary cause of non-adherence to surgical prophylaxis guidelines is exceeding the recommended duration of 24 h in 58% of instances [10].

In our pursuit of altering prescription patterns, our primary objective is to ensure the long-term sustainability of these changes while also fostering a deep conviction among physicians regarding the positive impact of these changes on patient wellbeing.

At HCB, our AMS program employs a strategy known as handshake stewardship, a strategy that has proven successful in other programs. Through this approach, we give prescribers a sense of ownership, effectively minimizing potential resistance from physicians. Simultaneously, this strategy has the potential to influence pharmacological management by making specialists aware that their prescription practices are being monitored and evaluated on an ongoing basis [30,31,32,33].

Prior to the implementation of the AMS, Ceftriaxone was commonly used as the antibiotic of choice for many surgeries. This same pattern has been demonstrated in other Latin American countries [10]. While this medication is widely used in hospitals, its improper use can stimulate the emergence of microorganisms harboring resistance genes such as extended-spectrum beta-lactamases (ESBLs). This has become a significant issue in the region, posing a serious threat to public health [3,34]. Our study results are consistent with other research that has reported similar outcomes. Implementing AMS has led to reductions in the utilization of third-generation cephalosporins, including Ceftriaxone, and a gradual upward utilization of Cefazolin (Figure 1) [24]. This pattern could be attributed to the collaborative efforts and concerted initiatives undertaken by various specialist groups [23]. In contrast to comparable studies, our research has revealed a relatively low utilization of vancomycin as a surgical prophylaxis agent [8,12,35].

Cumulative reports of antibiotic sensitivity were conducted at the HCB during the periods 2014–2015 and 2016–2017, which coincide with the development of our study. Significant decreases in ESBLs were observed among *E. coli* [36]. It is likely that the changes were influenced by actions implemented by the AMS for the optimal selection of surgical prophylaxis, along with other initiatives aimed at optimizing the use of antimicrobials. AMS initiatives like ours have demonstrated improvements in the resistance profile of microorganisms, as shown in a meta-analysis published in 2017 [37]. The analysis revealed a reduction in the incidence of multi-resistant Gram-negative bacilli (RR 0.49, 95% CI: 0.35–0.68), and Gram-negative bacilli with ESBL (RR 0.52; 95% CI: 0.27–0.98) [37].

The hospital has achieved a low incidence of surgical site infections, which is attributed to various factors, including the appropriate use of antibiotics. The use of antibiotics is a well-established practice supported by strong evidence for preventing the occurrence of infections [29]. This information could be useful for AMS teams of this region and for physicians who are concerned about changing their prescriptions for narrower-spectrum antibiotics, given the levels of antibiotic resistance in Latin America [16]. The incidence of infections declined during the COVID-19 pandemic, specifically in 2021, possibly due to the decrease in the number of procedures performed during this time.

Ensuring the successful adoption of AMS is paramount in tackling the escalating issue of antibiotic resistance within the region. Strengthening AMS in Latin America entails pivotal steps, including allocating additional resources and gaining a comprehensive understanding of the substantial challenges at hand. This endeavor encompasses evaluating the safety culture and acknowledging the human factors that exert influence on antibiotic prescribing practices [16]. By strategically addressing these factors, the region can bolster its efforts to curb antibiotic resistance and promote judicious antimicrobial use.

It is crucial to finely adjust the implemented interventions from the perspective of continuous quality improvement. Initially, this AMS directed education toward surgeons of various specialties. However, in numerous instances, anesthesiologists at this facility are responsible for prescribing prophylactics. Consequently, based on recommendations from the surgeons themselves, the scope was expanded to include these specialists. Additionally, as part of the conducted group educational sessions, the educational coverage was extended to encompass internists and intensivist physicians due to their pivotal role (in this specific hospital), in prescribing prophylactic antibiotics after conducting preoperative assessments on hospitalized patients, particularly those at high risk. These adjustments were pivotal in achieving the objectives of this AMS, underscoring the effectiveness of the continuous quality improvement approach in redefining effective actions [22].

Due to the time-intensive nature of conducting audits as performed in this specific study, we unfortunately lack quarterly results showcasing the incremental improvements in adherence to the clinical guidelines. To indirectly correlate compliance with clinical guidelines with a possible continuous improvement during the intervention period, Table 4 and Figure 1 shows how the trend in cefazolin consumption increased, while ceftriaxone consumption decreased over time.

The present study does not analyze the administration time of the prophylactic antibiotic. This limitation arises from the hospital’s model, which makes it challenging to obtain precise information on the exact timing of medication application. However, as of June 2022, a pharmacy was established in the surgical ward to monitor medication administration times. A further methodological limitation of this study is the lack of a calculated sample size for the periods under comparison. Rather, the analysis encompassed all surgeries conducted within the respective time frames. This could influence the results obtained. Another limitation is the long duration of this study, as multiple external factors, in addition to the implementation of the AMS, could influence the observed improvement in the selection of surgical prophylaxis. Some factors to consider may include the possibility that prescribers may have received information related to the appropriate prescribing of antibiotic prophylaxis for surgery from other institutions and the potential impact of new physicians on the post-AMS group.

Despite the factors mentioned earlier, the AMS program at this hospital is among the first of its kind in the country, and the rate of new doctors joining the hospital is low due to the absence of residency programs. Additionally, the development of AMS programs in the region is limited, the education related to this topic at the pre-graduate level is insufficient, and evidence suggests that antibiotic prophylaxis is a cause for concern in Latin America [10,16,19]. The inherent limitations of retrospective data collection, including potential bias and incomplete data, should be considered when interpreting our findings. Additionally, it is worth noting that our study was conducted at a relatively small, private hospital with 78 beds. This setting may introduce questions regarding the generalizability of our results to larger or differently structured healthcare institutions. However, even though we are a small hospital, we perform a significant number of surgeries, and are likely one of the top performers within the private healthcare system in Costa Rica.

## 4. Materials and Methods

### 4.1. Setting and Study Design

We conducted a single-center retrospective quasi-experimental design with patient matching at a 78-bed private hospital in San José, Costa Rica. Two study periods were defined: the pre-AMS period (January to June 2013) and the post-AMS period (June to December 2020). These two periods are related to the audit of all surgical procedures performed in the hospital for six months in each period. The pre-AMS audit demanded a significant investment of time and resources, rendering it impractical to conduct a re-audit during the implementation period, as no major changes were expected. The intervention period began in 2015 and continued for five years.

The intervention involved the implementing a surgical prophylaxis guideline. We conducted both group and individualized education sessions, progressively targeting specific medical specialties throughout the intervention. Furthermore, we shared educational materials, continuously monitored outcomes through performance indicators, and conducted both prospective and retrospective audits, providing individualized feedback based on our findings.

In this hospital, the surgeons perform mostly elective procedures, so there is not a significant difference in the types of surgeries throughout the year. The study included all patients who underwent a surgical procedure at the hospital during the designated timeframe and received antibiotic prophylaxis.

### 4.2. Data Collection

The information was obtained from the electronic clinical records of the hospital. The following were obtained: date, type of procedure, the physician in charge, antibiotic used as prophylaxis, and duration of prophylaxis.

### 4.3. Outcome Measures

The main outcome was to measure the difference in the proportion of the selection of optimal antibiotics for all surgical prophylaxis in the two periods of the study. An optimal selection was considered when the prescription complied with the antibiotics recommended in the previously mentioned guidelines, which are based on Bratzler’s guidelines [23]. Secondary outcomes included the proportion of surgical patients in the same period for whom the duration of antibiotic prophylaxis did not exceed 24 h following surgery (non-optimal duration).

Additional measures performed included monitoring of antibiotic consumption, adverse drug reactions, and incidence of surgical site infections. These secondary variables, due to their easy accessibility, were monitored throughout the established periods. Also, they served as indirect markers to assess the impact of the AMS over the years.

Antibiotic consumption was determined using the Anatomical Therapeutic Chemical/Defined Daily Dose (ATC/DDD) index [38]. Doses were normalized using DDD/1000 patient days. This parameter represents the overall consumption of HCB, it was not possible to determine the specific consumption in the surgical ward due to the limitations of the electronic system. The adverse effects related to medications and infections at the surgical site of the HCB were evaluated, searching for records associated with the prescriptions included in the study. The HCB Infection Control Committee provided reports of surgical site infections.

### 4.4. Statistical Analysis

Statistical methods such as the Chi-square and Fisher tests were employed to compare appropriate antibiotic selection and treatment duration. To assess antimicrobial consumption, an unpaired *t*-test was utilized for normally distributed data, while the nonparametric Mann–Whitney U test was used for data that lacked normal distribution. The data were subjected to a comprehensive statistical analysis using Excel and the most recent version of SPSS Software.

### 4.5. Ethics Approval and Consent to Participate

Ethical approval to conduct this study was obtained from the Scientific Ethical Committee of the University of Medical Sciences (CEC-UCIMED), approval date 2 June 2021, and reference number CEC-0312-2021. Written consent was not necessary for this study.

## 5. Conclusions

Implementing an AMS in the surgical ward of a Latin American hospital can lead to significant improvements in both the selection and duration of surgical antibiotic prophylaxis for cesarean deliveries and orthopedic surgeries. This may lead to a decrease in the use of ceftriaxone and a heightened preference for alternative agents like cefazolin. Importantly, the study found no noteworthy detrimental effects or increase in surgical site infections among patients who received improved antibiotic prophylaxis. This demonstrates the program’s success in optimizing antibiotic use without compromising patient safety.

## Figures and Tables

**Table 1:** Optimal selection of surgical prophylaxis in the pre-AMS and post-AMS periods according to the procedure.

Procedure	Pre-AMS *(N* = 1598)		Post-AMS (*N* = 841)		*p*-Value
	*N* (%)	Optimal Selection (%)	*N* (%)	Optimal Selection (%)	
Cesarean delivery	571 (35.7)	1.8	247 (29.4)	95.1	0.0001
Orthopedic	311 (19.5)	43.4	182 (21.6)	96.2	0.0001
Head and neck	103 (6.4)	31.1	112 (13.3)	28.6	0.6892
Urologic	107 (6.7)	32.7	46 (5.5)	93.5	0.0001
Hysterectomy	78 (4.9)	2.6	54 (6.4)	72.2	0.0001
Plastic surgery	95 (5.9)	26.3	31 (3.7)	93.5	0.0001
Biliary tract	61 (3.8)	75.4	33 (3.9)	90.9	0.0688
Other procedures *	272 (17.0)	18.4	136 (16.2)	64.7	0.0001

Note: AMS: Antimicrobial stewardship program. Other procedures *: small intestine, cardiac, gastro-duodenal tract, vascular, colorectal, thoracic, neurological, laparoscopic procedures, appendectomy for uncomplicated appendicitis, and various other unspecified procedures.

**Table 2:** Optimal duration of surgical prophylaxis in the pre-AMS and post-AMS periods according to the procedure.

Procedure	Pre-AMS (*N* = 1598)		Post-AMS (*N* = 841)		*p*-Value
	*N* (%)	Optimal Duration (%)	*N* (%)	Optimal Duration (%)	
Cesarean delivery	571 (35.7)	87.9	247 (29.4)	98.0	0.0001
Orthopedic	311 (19.5)	49.8	182 (21.6)	65.4	0.0008
Head and neck	103 (6.4)	60.2	112 (13.3)	64.3	0.5353
Urologic	107 (6.7)	53.3	46 (5.5)	54.3	0.9124
Hysterectomy	78 (4.9)	70.5	54 (6.4)	94.4	0.0007
Plastic surgery	95 (5.9)	66.3	31 (3.7)	77.4	0.2460
Biliary tract	61 (3.8)	72.1	33 (3.9)	84.8	0.1645
Other procedures *	272 (17.0)	61.0	136 (16.2)	69.9	0.0767

Note: AMS: Antimicrobial stewardship program. Other procedures *: small intestine, cardiac, gastro-duodenal tract, vascular, colorectal, thoracic, neurological, laparoscopic procedures, appendectomy for uncomplicated appendicitis, and various other unspecified procedures.

**Table 3:** Primary preoperative antibiotics selected as surgical prophylaxis in the pre-AMS and post-AMS periods.

Antibiotics Selected as Surgical Prophylaxis	Pre-AMS *N* (%)	Post-AMS *N* (%)	*p*-Value
Ceftriaxone	1168 (73.1)	106 (12.6)	0.0001
Cefazolin	0 (0)	476 (56.6)	-
Cephalothin	129 (8.1)	20 (2.4)	0.3628
Ampicillin Sulbactam	100 (6.3)	32 (3.4)	0.5353
Ampicillin	0 (0)	28 (3.3)	-
Amoxicillin Sulbactam	0 (0)	53 (6.3)	-
Other antibiotics *	198 (12.4)	126 (15.0)	0.5029

Note: AMS: Antimicrobial stewardship program. Other antibiotics *: Amikacin, ciprofloxacin, clindamycin, Ertapenem, gentamicin, levofloxacin, metronidazole, oxacillin, vancomycin.

**Table 4:** Consumption of antibiotics (DDD/1000 patient days) is most widely used in surgical prophylaxis using the DDD/1000 patient days method in the established periods.

Selected Antibiotic	Pre-AMS (DDD/1000 Patient Days)	Post-AMS (DDD/1000 Patient Days)	Magnitude of Difference	*p*-Value
Ceftriaxone	217.7	139.8	▼ 77.9	0.0190
Cefazolin	14.9	153.3	▲ 149.6	0.0210

Note: AMS: Antimicrobial stewardship program. DDD: defined daily dose. ▼ decreases. ▲ increases.

## Data Availability

The data are not available due to the confidentiality established in the legislation of this country.
